# Bis(μ-di­phenyl­phosphan­yl)bis­[(tri­methyl­phosphane)cobalt(I)](*Co*—*Co*)

**DOI:** 10.1107/S1600536813027384

**Published:** 2013-10-16

**Authors:** Robert Beck, Hans-Friedrich Klein

**Affiliations:** a Eduard-Zintl-Institut, Darmstadt University of Technology, 64287 Darmstadt, Germany

## Abstract

The title compound, [Co_2_{P(C_6_H_5_)_2_}_2_(C_3_H_9_P)_4_], was obtained by the addition of di­phenyl­phosphane to a solution of Co(CH_3_)(C_3_H_9_P)_4_. The dinuclear complex mol­ecule exhibits inversion symmetry with the inversion centre located between the two Co^I^ atoms. The short Co—Co distance of 2.3670 (8) Å lies within the range of metal–metal double bonds. As a result of inversion symmetry, the four-membered Co_2_P_2_ core is rigorously planar, and the two bridging P(C_6_H_5_)_2_-ligands and the terminal C_3_H_9_P ligands are arranged in a pseudo-tetra­hedral fashion about the Co^I^ atom.

## Related literature

For related homobimetallic cobalt complexes, see: Harley *et al.* (1983 ▶); Jones *et al.* (1983 ▶); Winter­halter *et al.* (2001 ▶): For related salt metathesis reactions, see: Klein *et al.* (1988 ▶, 2003 ▶); Klein & Karsch (1975 ▶).
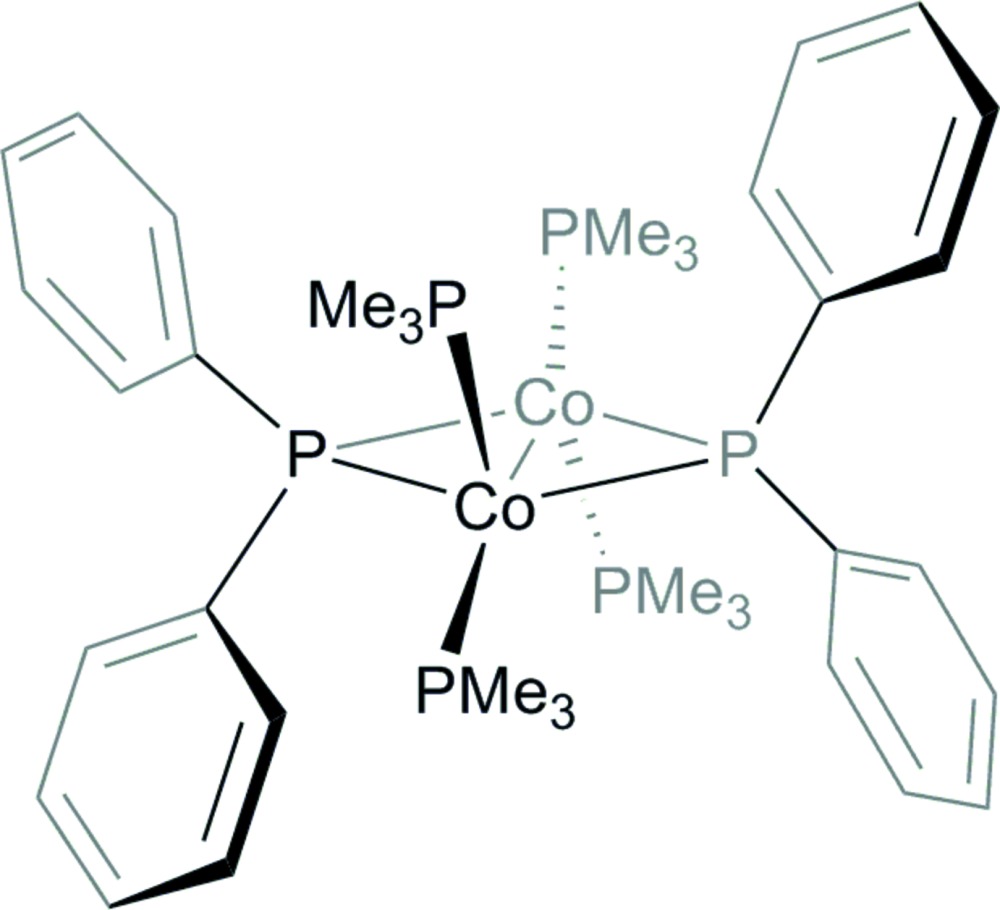


## Experimental

### 

#### Crystal data

[Co_2_(C_12_H_10_P)_2_(C_3_H_9_P)_4_]
*M* *_r_* = 792.49
Monoclinic, 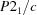

*a* = 10.318 (2) Å
*b* = 19.262 (4) Å
*c* = 10.721 (2) Å
β = 113.32 (3)°
*V* = 1956.8 (7) Å^3^
*Z* = 2
Mo *K*α radiation
μ = 1.12 mm^−1^
*T* = 173 K
0.12 × 0.10 × 0.08 mm

#### Data collection

Bruker APEX CCD diffractometer
Absorption correction: multi-scan (*SADABS*; Sheldrick, 2003 ▶) *T*_min_ = 0.912, *T*_max_ = 0.940
37952 measured reflections
5413 independent reflections
3251 reflections with *I* > 2σ(*I*)
*R*_int_ = 0.062

#### Refinement

*R*[*F* ^2^ > 2σ(*F* ^2^)] = 0.037
*wR*(*F* ^2^) = 0.083
*S* = 0.81
5413 reflections
205 parameters
H-atom parameters constrained
Δρ_max_ = 0.45 e Å^−3^
Δρ_min_ = −0.86 e Å^−3^

### 

Data collection: *SMART* (Bruker, 2001 ▶); cell refinement: *SAINT* (Bruker, 2001 ▶); data reduction: *SAINT*; program(s) used to solve structure: *SHELXS97* (Sheldrick, 2008 ▶); program(s) used to refine structure: *SHELXL97* (Sheldrick, 2008 ▶); molecular graphics: *ORTEP-3 for Windows* (Farrugia, 2012 ▶); software used to prepare material for publication: *OLEX2* (Dolomanov *et al.*, 2009 ▶).

## Supplementary Material

Crystal structure: contains datablock(s) I. DOI: 10.1107/S1600536813027384/wm2765sup1.cif

Structure factors: contains datablock(s) I. DOI: 10.1107/S1600536813027384/wm2765Isup2.hkl

Additional supplementary materials:  crystallographic information; 3D view; checkCIF report

## Figures and Tables

**Fig. 1.**
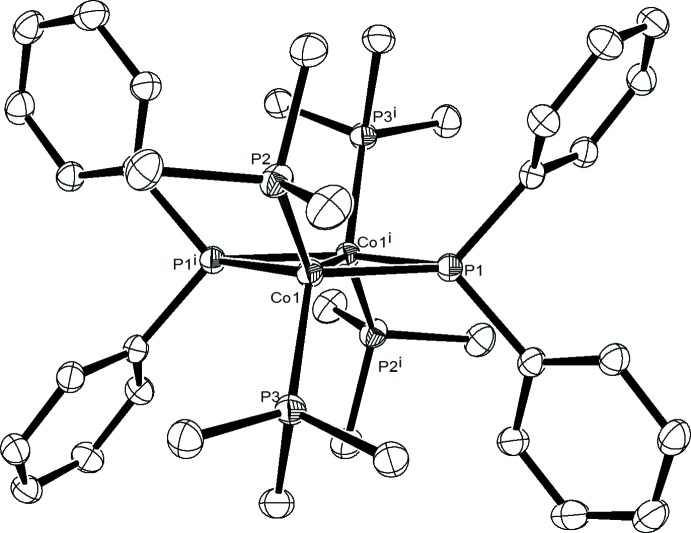
The molecular structure of compound (I), with atom labels and thermal displacement parameters of the atoms drawn at the 50% probability level for non-H atoms; H atoms were omitted for clarity. Symmetry code (i) -x, 1 - y, -z indicates symmetry-related atoms generated by a crystallographic inversion centre.

**Fig. 2.**
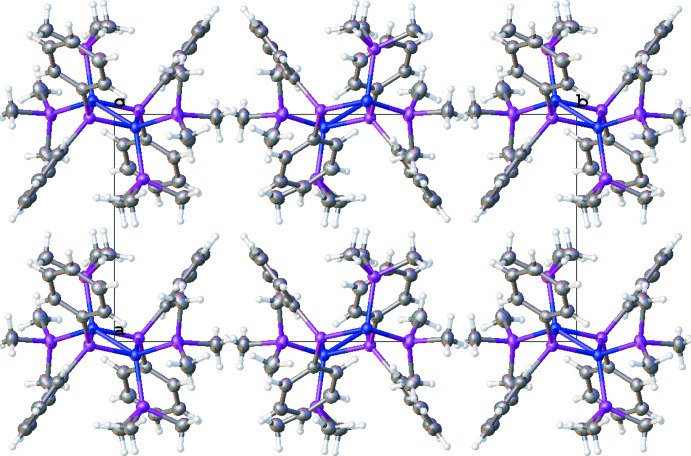
Crystal packing of compound (I) viewed along [001].

## supplementary crystallographic information

### 1. Comment

The synthesis of bimetallic complexes with bridging anionic P*R*_2_ ligands (*R* = aryl or alkyl) are long time established (Harley *et al.*, 1983), and numerous variants of synthetic approaches were developed (Jones *et al.*, 1983). During the course of our investigations into the chemistry of cyclometallation of triphenylphosphane derivatives (Winterhalter *et al.*, 2001), we isolated, structurally and spectroscopically characterized a series of *ortho*-metallated cobalt complexes (Klein *et al.*, 2003). Using previous method allowed to synthesize a related bimetallic cobalt complex *via* salt metathesis of CoCl(PMe_3_)_3_ with LiPPMe_2_ in high yield (Klein *et al.*, 1988).

The molecular structure of the bimetallic title complex, (I), [Co(C_3_H_9_P)_2_(µ_2_-P(C_6_H_5_)_2_)]_2_, is shown in Fig. 1. The complex exhibits a crystallographically imposed inversion centre in the middle of the molecule. Each Co^I^ atom has a distorted tetrahedral coordination geometry with a short Co—Co distance of 2.3670 (8) Å, slightly longer than found for other Co═Co distances of homobimetallic cobalt complexes. Each set of terminal ligands is *trans* with respect to the Co═Co bond. Both bridging µ_2_-PPh_2_ and PMe_3_-ligands are bent away from the central Co_2_P_2_ core with the distortion from idealized tetrahedral geometry being greater for the larger PPh_2_ group with 115.13 (3)°.

The crystal packing of compound (I) can be described as being composed of rods of single molecules stacked along [100] and [001], with the Co_2_P_2_ cores arranged in alternating directions (Fig. 2).

### 2. Experimental

Standard vacuum techniques were used in manipulations of volatile and air sensitive material. Literature methods were applied for the preparation of Co(CH_3_)(PMe_3_)_4_ (Klein & Karsch, 1975). Other chemicals were used as purchased. The title compound bis(µ_2_-diphenylphosphino)tetrakis(trimethylphosphane) dicobalt(I) was synthesized by combining stoichiometric amounts of diphenylphosphane (118 mg, 0.63 mmol) in 20 ml of *n*-pentane at 203 K with a sample of Co(CH_3_)(PMe_3_)_4_ (240 mg, 0.63 mmol) in 20 ml of *n*-pentane, effecting a change of color from red to dark brown. After warm-up, the mixture was kept stirring at 293 K for 16 h, and then the volatiles were removed *in vacuo* to give a dark brown, waxy solid. This was dissolved in a mixture of 10 ml of *n*-pentane / diethyl ether (3:1) and crystallized at 253 K to give brown rhombic crystals, which were suitable for X-ray diffraction. Isolated yield 145 mg (58%); m. p. 391–393 K (dec.). ^1^H NMR (300 MHz, THF-d_8_, 293 K, p.p.m.): δ = 0.88 (*s*(br), 36H, PCH_3_); 7.03 - 7.11 (m, 12H, Ar—H); 7.51 – 7.56 (m, 8H, Ar—H); - ^31^P{^1^H} NMR (121 MHz, THF-d_8_, 297 K, p.p.m.): δ = 4.66 (*s*(br), 4P, PCH_3_), 12.3 (*s*(br), 2P, PPh_2_). Anal. Calcd. for C_36_H_56_Co_2_P_6_ (792.5): C, 54.56; H, 7.12; P, 23.45: Found: C, 54.88; H, 6.72; P 23.98%.

### 3. Refinement

H atoms were fixed geometrically and treated as riding on their parent atoms with C—H = 0.93 Å (aromatic) and 0.96 Å (methyl), and with *U*_iso_(H) = 1.2 (1.5 for methyl groups) times *U*_eq_(C).

### Crystal data

**Table:**

[Co_2_(C_12_H_10_P)_2_(C_3_H_9_P)_4_]	*F*(000) = 832
*M**_r_* = 792.49	*D*_x_ = 1.345 Mg m^−^^3^
Monoclinic, *P*2_1_/*c*	Mo *K*α radiation, λ = 0.71073 Å
*a* = 10.318 (2) Å	Cell parameters from 864 reflections
*b* = 19.262 (4) Å	θ = 3.0–26.0°
*c* = 10.721 (2) Å	µ = 1.12 mm^−^^1^
β = 113.32 (3)°	*T* = 173 K
*V* = 1956.8 (7) Å^3^	Block, brown
*Z* = 2	0.12 × 0.10 × 0.08 mm

### Data collection

**Table:**

Bruker APEX CCD diffractometer	5413 independent reflections
Radiation source: fine-focus sealed tube	3251 reflections with *I* > 2σ(*I*)
Graphite monochromator	*R*_int_ = 0.062
phi and ω scans	θ_max_ = 29.5°, θ_min_ = 2.1°
Absorption correction: multi-scan (*SADABS*; Sheldrick, 2003)	*h* = −14→14
*T*_min_ = 0.912, *T*_max_ = 0.940	*k* = −26→26
37952 measured reflections	*l* = −14→12

### Refinement

**Table:**

Refinement on *F*^2^	Primary atom site location: structure-invariant direct methods
Least-squares matrix: full	Secondary atom site location: difference Fourier map
*R*[*F*^2^ > 2σ(*F*^2^)] = 0.037	Hydrogen site location: inferred from neighbouring sites
*wR*(*F*^2^) = 0.083	H-atom parameters constrained
*S* = 0.81	*w* = 1/[σ^2^(*F*_o_^2^) + (0.0484*P*)^2^] where *P* = (*F*_o_^2^ + 2*F*_c_^2^)/3
5413 reflections	(Δ/σ)_max_ = 0.001
205 parameters	Δρ_max_ = 0.45 e Å^−^^3^
0 restraints	Δρ_min_ = −0.86 e Å^−^^3^

### Special details

**Table:**

Geometry. All e.s.d.'s (except the e.s.d. in the dihedral angle between two l.s. planes) are estimated using the full covariance matrix. The cell e.s.d.'s are taken into account individually in the estimation of e.s.d.'s in distances, angles and torsion angles; correlations between e.s.d.'s in cell parameters are only used when they are defined by crystal symmetry. An approximate (isotropic) treatment of cell e.s.d.'s is used for estimating e.s.d.'s involving l.s. planes.
Refinement. Refinement of *F*^2^ against ALL reflections. The weighted *R*-factor *wR* and goodness of fit *S* are based on *F*^2^, conventional *R*-factors *R* are based on *F*, with *F* set to zero for negative *F*^2^. The threshold expression of *F*^2^ > σ(*F*^2^) is used only for calculating *R*-factors(gt) *etc*. and is not relevant to the choice of reflections for refinement. *R*-factors based on *F*^2^ are statistically about twice as large as those based on *F*, and *R*- factors based on ALL data will be even larger.

### Fractional atomic coordinates and isotropic or equivalent isotropic displacement parameters (Å^2^)

**Table:**

	*x*	*y*	*z*	*U*_iso_*/*U*_eq_	
Co1	0.05854 (3)	0.453786 (15)	0.07344 (3)	0.02705 (8)	
P1	0.01598 (6)	0.55315 (3)	0.14726 (6)	0.02866 (12)	
P2	0.00076 (7)	0.36275 (3)	0.15980 (6)	0.03557 (15)	
P3	0.28562 (6)	0.43802 (3)	0.16358 (6)	0.03235 (14)	
C1	0.1571 (2)	0.61095 (11)	0.2607 (2)	0.0314 (5)	
C2	0.2519 (2)	0.64148 (12)	0.2142 (3)	0.0379 (5)	
H2	0.2402	0.6342	0.1227	0.045*	
C3	0.3630 (3)	0.68225 (13)	0.2982 (3)	0.0461 (6)	
H3	0.4249	0.7034	0.2634	0.055*	
C4	0.3839 (3)	0.69221 (14)	0.4325 (3)	0.0479 (7)	
H4	0.4611	0.7193	0.4909	0.057*	
C5	0.2914 (3)	0.66245 (14)	0.4807 (3)	0.0448 (6)	
H5	0.3049	0.6692	0.5728	0.054*	
C6	0.1783 (3)	0.62262 (12)	0.3958 (2)	0.0360 (5)	
H6	0.1146	0.6031	0.4303	0.043*	
C7	−0.1106 (2)	0.56800 (12)	0.2273 (2)	0.0304 (5)	
C8	−0.1900 (2)	0.62912 (12)	0.1978 (2)	0.0347 (5)	
H8	−0.1728	0.6637	0.1430	0.042*	
C9	−0.2939 (3)	0.64003 (13)	0.2478 (3)	0.0385 (5)	
H9	−0.3478	0.6816	0.2259	0.046*	
C10	−0.3189 (3)	0.59079 (13)	0.3289 (3)	0.0397 (6)	
H10	−0.3916	0.5977	0.3609	0.048*	
C11	−0.2371 (3)	0.53124 (14)	0.3632 (3)	0.0412 (6)	
H11	−0.2517	0.4977	0.4213	0.049*	
C12	−0.1339 (3)	0.52056 (13)	0.3129 (2)	0.0356 (5)	
H12	−0.0779	0.4797	0.3378	0.043*	
C13	−0.1791 (3)	0.35558 (15)	0.1568 (3)	0.0485 (7)	
H13A	−0.1993	0.3965	0.2005	0.073*	
H13B	−0.1856	0.3137	0.2059	0.073*	
H13C	−0.2478	0.3528	0.0625	0.073*	
C14	0.0125 (3)	0.27953 (13)	0.0821 (3)	0.0465 (6)	
H14A	−0.0508	0.2801	−0.0143	0.070*	
H14B	−0.0151	0.2419	0.1280	0.070*	
H14C	0.1097	0.2721	0.0909	0.070*	
C15	0.1016 (3)	0.34134 (16)	0.3393 (3)	0.0525 (7)	
H15A	0.2003	0.3327	0.3546	0.079*	
H15B	0.0617	0.2997	0.3629	0.079*	
H15C	0.0965	0.3802	0.3962	0.079*	
C16	0.3665 (3)	0.35193 (14)	0.1710 (3)	0.0450 (6)	
H16A	0.3405	0.3213	0.2304	0.068*	
H16B	0.4694	0.3567	0.2071	0.068*	
H16C	0.3327	0.3320	0.0796	0.068*	
C17	0.3846 (3)	0.48700 (14)	0.0843 (3)	0.0419 (6)	
H17A	0.3540	0.4730	−0.0110	0.063*	
H17B	0.4857	0.4775	0.1323	0.063*	
H17C	0.3673	0.5368	0.0890	0.063*	
C18	0.3801 (3)	0.46387 (13)	0.3413 (2)	0.0401 (6)	
H18A	0.3655	0.5135	0.3509	0.060*	
H18B	0.4811	0.4547	0.3693	0.060*	
H18C	0.3442	0.4373	0.3988	0.060*	

### Atomic displacement parameters (Å^2^)

**Table:**

	*U*^11^	*U*^22^	*U*^33^	*U*^12^	*U*^13^	*U*^23^
Co1	0.02893 (15)	0.02544 (14)	0.02803 (15)	−0.00128 (12)	0.01259 (11)	−0.00020 (12)
P1	0.0307 (3)	0.0278 (3)	0.0289 (3)	−0.0012 (2)	0.0132 (2)	−0.0022 (2)
P2	0.0428 (4)	0.0298 (3)	0.0350 (3)	−0.0054 (2)	0.0164 (3)	0.0019 (2)
P3	0.0299 (3)	0.0338 (3)	0.0320 (3)	0.0013 (2)	0.0108 (2)	−0.0002 (2)
C1	0.0301 (11)	0.0251 (10)	0.0356 (12)	0.0023 (8)	0.0095 (10)	−0.0021 (9)
C2	0.0325 (12)	0.0364 (12)	0.0439 (14)	−0.0014 (9)	0.0142 (11)	−0.0024 (11)
C3	0.0336 (13)	0.0367 (13)	0.0651 (18)	−0.0008 (10)	0.0162 (13)	0.0016 (12)
C4	0.0344 (13)	0.0395 (14)	0.0557 (17)	0.0006 (11)	0.0028 (12)	−0.0098 (12)
C5	0.0376 (13)	0.0461 (14)	0.0396 (14)	0.0038 (11)	0.0035 (11)	−0.0097 (11)
C6	0.0323 (12)	0.0355 (12)	0.0353 (12)	0.0018 (9)	0.0083 (10)	−0.0030 (10)
C7	0.0317 (11)	0.0327 (11)	0.0259 (11)	−0.0023 (9)	0.0105 (9)	−0.0054 (9)
C8	0.0382 (13)	0.0321 (11)	0.0350 (12)	−0.0017 (9)	0.0156 (10)	−0.0035 (9)
C9	0.0362 (13)	0.0383 (13)	0.0404 (13)	0.0039 (10)	0.0145 (11)	−0.0044 (10)
C10	0.0339 (12)	0.0505 (15)	0.0374 (13)	−0.0002 (11)	0.0171 (11)	−0.0045 (11)
C11	0.0439 (14)	0.0472 (15)	0.0380 (13)	0.0008 (11)	0.0220 (11)	0.0054 (11)
C12	0.0361 (12)	0.0365 (12)	0.0358 (12)	0.0020 (10)	0.0159 (10)	0.0018 (10)
C13	0.0543 (17)	0.0484 (15)	0.0512 (16)	−0.0161 (12)	0.0297 (14)	−0.0055 (12)
C14	0.0529 (16)	0.0309 (12)	0.0516 (16)	−0.0010 (11)	0.0164 (13)	0.0017 (11)
C15	0.0659 (18)	0.0481 (16)	0.0425 (15)	−0.0118 (14)	0.0204 (14)	0.0074 (12)
C16	0.0410 (14)	0.0445 (14)	0.0438 (15)	0.0086 (11)	0.0106 (12)	−0.0019 (11)
C17	0.0334 (13)	0.0527 (15)	0.0395 (13)	−0.0042 (11)	0.0144 (11)	−0.0033 (12)
C18	0.0363 (12)	0.0437 (14)	0.0374 (13)	0.0023 (11)	0.0115 (10)	−0.0009 (11)

### Geometric parameters (Å, º)

**Table:**

Co1—Co1^i^	2.3670 (8)	C8—H8	0.9500
Co1—P1^i^	2.1835 (9)	C8—C9	1.391 (3)
Co1—P1	2.1812 (7)	C9—H9	0.9500
Co1—P2	2.1735 (7)	C9—C10	1.378 (4)
Co1—P3	2.1734 (9)	C10—H10	0.9500
P1—Co1^i^	2.1835 (9)	C10—C11	1.385 (4)
P1—C1	1.854 (2)	C11—H11	0.9500
P1—C7	1.847 (2)	C11—C12	1.387 (3)
P2—C13	1.849 (3)	C12—H12	0.9500
P2—C14	1.833 (3)	C13—H13A	0.9800
P2—C15	1.836 (3)	C13—H13B	0.9800
P3—C16	1.844 (3)	C13—H13C	0.9800
P3—C17	1.829 (3)	C14—H14A	0.9800
P3—C18	1.833 (3)	C14—H14B	0.9800
C1—C2	1.392 (3)	C14—H14C	0.9800
C1—C6	1.394 (3)	C15—H15A	0.9800
C2—H2	0.9500	C15—H15B	0.9800
C2—C3	1.388 (3)	C15—H15C	0.9800
C3—H3	0.9500	C16—H16A	0.9800
C3—C4	1.383 (4)	C16—H16B	0.9800
C4—H4	0.9500	C16—H16C	0.9800
C4—C5	1.377 (4)	C17—H17A	0.9800
C5—H5	0.9500	C17—H17B	0.9800
C5—C6	1.392 (3)	C17—H17C	0.9800
C6—H6	0.9500	C18—H18A	0.9800
C7—C8	1.397 (3)	C18—H18B	0.9800
C7—C12	1.382 (3)	C18—H18C	0.9800
			
P1—Co1—Co1^i^	57.21 (2)	C9—C8—C7	120.8 (2)
P1^i^—Co1—Co1^i^	57.11 (2)	C9—C8—H8	119.6
P1—Co1—P1^i^	114.32 (2)	C8—C9—H9	119.9
P2—Co1—Co1^i^	137.42 (3)	C10—C9—C8	120.3 (2)
P2—Co1—P1^i^	111.96 (3)	C10—C9—H9	119.9
P2—Co1—P1	115.13 (3)	C9—C10—H10	120.3
P3—Co1—Co1^i^	125.27 (3)	C9—C10—C11	119.4 (2)
P3—Co1—P1^i^	109.18 (4)	C11—C10—H10	120.3
P3—Co1—P1	107.32 (3)	C10—C11—H11	120.0
P3—Co1—P2	97.30 (3)	C10—C11—C12	120.0 (2)
Co1—P1—Co1^i^	65.68 (2)	C12—C11—H11	120.0
C1—P1—Co1	123.13 (7)	C7—C12—C11	121.5 (2)
C1—P1—Co1^i^	126.59 (8)	C7—C12—H12	119.2
C7—P1—Co1^i^	120.15 (7)	C11—C12—H12	119.2
C7—P1—Co1	125.83 (7)	P2—C13—H13A	109.5
C7—P1—C1	96.80 (10)	P2—C13—H13B	109.5
C13—P2—Co1	119.71 (10)	P2—C13—H13C	109.5
C14—P2—Co1	115.68 (10)	H13A—C13—H13B	109.5
C14—P2—C13	99.98 (13)	H13A—C13—H13C	109.5
C14—P2—C15	99.68 (14)	H13B—C13—H13C	109.5
C15—P2—Co1	119.38 (10)	P2—C14—H14A	109.5
C15—P2—C13	98.61 (14)	P2—C14—H14B	109.5
C16—P3—Co1	122.34 (9)	P2—C14—H14C	109.5
C17—P3—Co1	115.06 (9)	H14A—C14—H14B	109.5
C17—P3—C16	99.05 (13)	H14A—C14—H14C	109.5
C17—P3—C18	100.24 (12)	H14B—C14—H14C	109.5
C18—P3—Co1	117.43 (9)	P2—C15—H15A	109.5
C18—P3—C16	98.85 (12)	P2—C15—H15B	109.5
C2—C1—P1	119.95 (18)	P2—C15—H15C	109.5
C2—C1—C6	117.4 (2)	H15A—C15—H15B	109.5
C6—C1—P1	122.54 (19)	H15A—C15—H15C	109.5
C1—C2—H2	119.2	H15B—C15—H15C	109.5
C3—C2—C1	121.6 (3)	P3—C16—H16A	109.5
C3—C2—H2	119.2	P3—C16—H16B	109.5
C2—C3—H3	119.9	P3—C16—H16C	109.5
C4—C3—C2	120.2 (3)	H16A—C16—H16B	109.5
C4—C3—H3	119.9	H16A—C16—H16C	109.5
C3—C4—H4	120.4	H16B—C16—H16C	109.5
C5—C4—C3	119.2 (2)	P3—C17—H17A	109.5
C5—C4—H4	120.4	P3—C17—H17B	109.5
C4—C5—H5	119.7	P3—C17—H17C	109.5
C4—C5—C6	120.7 (3)	H17A—C17—H17B	109.5
C6—C5—H5	119.7	H17A—C17—H17C	109.5
C1—C6—H6	119.5	H17B—C17—H17C	109.5
C5—C6—C1	120.9 (2)	P3—C18—H18A	109.5
C5—C6—H6	119.5	P3—C18—H18B	109.5
C8—C7—P1	119.00 (18)	P3—C18—H18C	109.5
C12—C7—P1	123.14 (18)	H18A—C18—H18B	109.5
C12—C7—C8	117.8 (2)	H18A—C18—H18C	109.5
C7—C8—H8	119.6	H18B—C18—H18C	109.5

Symmetry code: (i) −*x*, −*y*+1, −*z*.
